# SorghumBase: a web-based portal for sorghum genetic information and community advancement

**DOI:** 10.1007/s00425-022-03821-6

**Published:** 2022-01-11

**Authors:** Nicholas Gladman, Andrew Olson, Sharon Wei, Kapeel Chougule, Zhenyuan Lu, Marcela Tello-Ruiz, Ivar Meijs, Peter Van Buren, Yinping Jiao, Bo Wang, Vivek Kumar, Sunita Kumari, Lifang Zhang, John Burke, Junping Chen, Gloria Burow, Chad Hayes, Yves Emendack, Zhanguo Xin, Doreen Ware

**Affiliations:** 1 Cold Spring Harbor Laboratory, Cold Spring Harbor, NY 11724 USA; 2 Plant Stress and Germplasm Development Unit, Cropping Systems Research Laboratory, U.S. Department of Agriculture-Agricultural Research Service, Lubbock, TX 79415 USA; 3 Department of Plant and Soil Science, Institute of Genomics for Crop Abiotic Stress Tolerance, Texas Tech University, Lubbock, TX 79409 USA; 4 U.S. Department of Agriculture-Agricultural Research Service, NEA Robert W. Holley Center for Agriculture and Health, Cornell University, Ithaca, NY 14853 USA

**Keywords:** Genomic, Genetic, Molecular, Breeding, Resource, Sorghum

## Abstract

**Main conclusion:**

SorghumBase provides a community portal that integrates genetic, genomic, and breeding resources for sorghum germplasm improvement.

**Abstract:**

Public research and development in agriculture rely on proper data and resource sharing within stakeholder communities. For plant breeders, agronomists, molecular biologists, geneticists, and bioinformaticians, centralizing desirable data into a user-friendly hub for crop systems is essential for successful collaborations and breakthroughs in germplasm development. Here, we present the SorghumBase web portal (https://www.sorghumbase.org), a resource for the sorghum research community. SorghumBase hosts a wide range of sorghum genomic information in a modular framework, built with open-source software, to provide a sustainable platform. This initial release of SorghumBase includes: (1) five sorghum reference genome assemblies in a pan-genome browser; (2) genetic variant information for natural diversity panels and ethyl methanesulfonate (EMS)-induced mutant populations; (3) search interface and integrated views of various data types; (4) links supporting interconnectivity with other repositories including genebank, QTL, and gene expression databases; and (5) a content management system to support access to community news and training materials. SorghumBase offers sorghum investigators improved data collation and access that will facilitate the growth of a robust research community to support genomics-assisted breeding.

**Supplementary Information:**

The online version contains supplementary material available at 10.1007/s00425-022-03821-6.

## Background

Sorghum [*Sorghum bicolor* (L.) Moench], domesticated in Northern Africa ~ 6000 years ago, comprises five major botanical races: bicolor, durra, guinea, caudatum, and kafir (Wet et al. 1967; Dillon et al. 2007). A C_4_ grass crop with tolerances to drought, heat, low nutrients, and high-salt conditions (Doumbia et al. 1993, 1998; Leiser et al. 2014; Hufnagel et al. 2014; Tack et al. 2017; Ochieng et al. 2021); sorghum is the fifth most important crop worldwide relating to acreage and production. Sorghum cultivars are grown for grain, silage, biomass, or syrup production. Historically, sorghum was predominantly used as feed and fodder for livestock, but since the mid-2010s, the amount used for the consumer food industry has risen > 250% (https://www.sorghumcheckoff.com/resources/research). Since the 1960s, global production of sorghum has increased, even while dedicated land use has decreased (http://www.fao.org/faostat/en/#data).

Sorghum is a useful model for crop research due to its compact genome; the first completely sequenced reference genome, BTx623, is ~ 730 Mb (Paterson et al. 2009; McCormick et al. 2018; Cooper et al. 2019). Sorghum shares functional genomic capabilities of agricultural plant systems such as maize, but compared to maize, most domesticated sorghum lines have fewer deleterious mutations relative to wild landraces (Lozano et al. 2021), likely due to a hermaphroditic inflorescence and the higher incidence of selfing (> 80%) (Djè et al. 2004; Barnaud et al. 2008) during germplasm selection, conversion, and improvement (Lai et al. 2018).

As climate change progresses and arable land becomes limited (Intergovernmental Panel on Climate Change 2014), sorghum serves as an essential crop for addressing the challenge of feeding an ever-increasing world population. Global germplasm repositories such as the International Crops Research Institute for the Semi-Arid Tropics (ICRISAT; http://genebank.icrisat.org/) and the Germplasm Resource Information Network (GRIN; https://npgsweb.ars-grin.gov/gringlobal) house tens of thousands of domesticated sorghum cultivars and wild landraces comprising vast genetic potential for use in yield improvement in diverse farming operations. Core germplasm collections such as the Sorghum Association Panel (Casa et al. 2008), Bioenergy Association Panel (Brenton et al. 2016), and Nested Association Mapping population (Bouchet et al. 2017; Boatwright et al. 2021; Perumal et al. 2021) were created to enable breeders and geneticists to dissect the molecular underpinnings of traits including grain yield, nutrient use efficiency (Shakoor et al. 2016), and disease resistance (Cuevas et al. 2019). Complementing these resources, the genomes of many important lines have been sequenced (BTx623, Rio, Tx2783, RTx436, and RTx430), with dozens more on the way, constituting a sorghum pan-genome dataset with myriad potential applications.

Here, we describe SorghumBase (https://www.sorghumbase.org), a web-based community resource designed as an access point for the sorghum genomic/molecular research and breeding community.

## Introduction to SorghumBase portal

SorghumBase is a genomic resource for the sorghum community, stably funded by the United States Department of Agriculture (USDA) just like MaizeGDB (Woodhouse et al. 2021), GrainGenes (Blake et al. 2019), and Soybase (Grant et al. 2010). This resource was developed to support stewardship and sharing of emergent sorghum genomic and genetic data, with the goal of accelerating knowledge accumulation associated with high-value traits by harnessing genomic, genetic, and functional information generated by the sorghum research community. SorghumBase follows findability, accessibility, interoperability, and reusability (FAIR) guidelines (Wilkinson et al. 2016). The foundations of SorghumBase focus on improving management of genomic related data sets, while promoting standards, open access to data, and information sharing with the broader community. The top priority for SorghumBase is stewardship of sorghum reference genomes: a cornerstone for accessing and characterizing allelic variation underlying important agronomic traits. The portal uses Ensembl and Gramene open-source software (Kersey et al. 2018; Tello-Ruiz et al. 2021), representing more than 23 years of development of data models, workflows, robust visualizations, and application interfaces (APIs).

## Sorghum genomes

The first public release includes five public reference assemblies along with the community annotations for BTx623 (McCormick et al. 2018), Tx2783 (Wang et al. 2021), RTx430 (Deschamps et al. 2018), RTx436 (Wang et al. 2021), and Rio (Cooper et al. 2019) (Table 1). For each genome, information on the assembly method and gene structural annotations is available from the home page of its individual genome browser. No standard nomenclature has yet been established for sorghum genes, transcripts, and proteins; instead, each community project uses its own naming assignments. SorghumBase stores reference genome assemblies, gene structures, and functional annotations of the genomes in an Ensembl genome core database. Ensembl data models and APIs have specific requirements, in agreement with International Nucleotide Sequence Database Collaboration (INSDC) gene annotation standards, for stable database identifiers that sometimes conflict with or require changes to existing community annotations. For example, Phytozome (Goodstein et al. 2012) has historically given sorghum genes names like ‘Sobic.004G141800’, which conflicts with INSDC standards. SorghumBase and EnsemblPlants (Howe et al. 2020) resolve this problem by assigning Phytozome names as synonyms while storing compliant names as the gene stable ID in the database. The Phytozome gene ID above is stored as ‘SORBI_3004G141800’, where ‘SORBI_3’ represents the species or germplasm name (SORBI for *Sorghum bicolor*) and assembly version. The rest of the identifier includes the chromosome on which the gene is located (004), followed by the identifier ‘G’ for gene and then a locus index based on the sequential order of loci on the chromosome (141,800). The project will work with the sorghum community to ensure that sorghum genome assemblies are in the correct format to support accessioning by one of the archives in the INSDC (http://www.insdc.org), e.g., the European Nucleotide Archive (ENA) from EMBL-EBI or the NCBI databases.

**Table 1:** Current pan-genome content and annotations

	BTx623 v3.1	Tx2783	RTx436	TX430 v2	Rio v2.1
Gene count	34,118	29,612	29,265	36,937	35,490
Gene length (ave)	3714	3833	3900	3252	3322
Gene length (median)	2824	2888	2917	2217	2469
Exon count	154,042	147,196	146,857	163,854	163,465
Exon length (ave)	449	357	353	348	353
Exon length (median)	176.5	172	171	174	172
Intron count	119,924	117,584	117,592	126,917	127,975
Intron length (ave)	454	512	514	483	467
Intron length (median)	142	150	150	147	146
CDS count	47,110	35,998	41,713	49,928	41,048
CDS length (ave)*	842	980	838	858	964
CDS length (median)*	981	1005	1002	981	939
Peptide count	47,110	35,998	41,713	49,928	41,048
Peptide length (ave)*	281	327	279	286	321
Peptide length (median)*	327	335	334	327	313
Five_prime_UTR count	25,100	32,455	30,983	22,796	30,096
Five_prime_UTR length (ave)	484	197	196	225	202
Five_prime_UTR length (median)	207	147	143	171	144
Three_prime_UTR count	26,660	29,444	29,109	22,761	30,239
Three_prime_UTR length (ave)	653	371	374	399	396
Three_prime_UTR length (median)	356	318	314	339	339
Exons per transcript (ave)	4.5	5	5	4.4	4.6
Single-exon gene count (pct)	8467 (24.8)	6783 (22.9)	6585 (22.5)	11,854 (32.1)	8128 (22.9)

Genome assembly and annotation descriptions of BTx623, Tx2783, RTx436, Tx430, and Rio. All currently available for analysis on SorghumBase

*Note: longest_CDS_transcript_used_for_calculation_of_CDS_and_Protein_length_statistics

## Phylogenetic gene trees

**Fig. 1:**
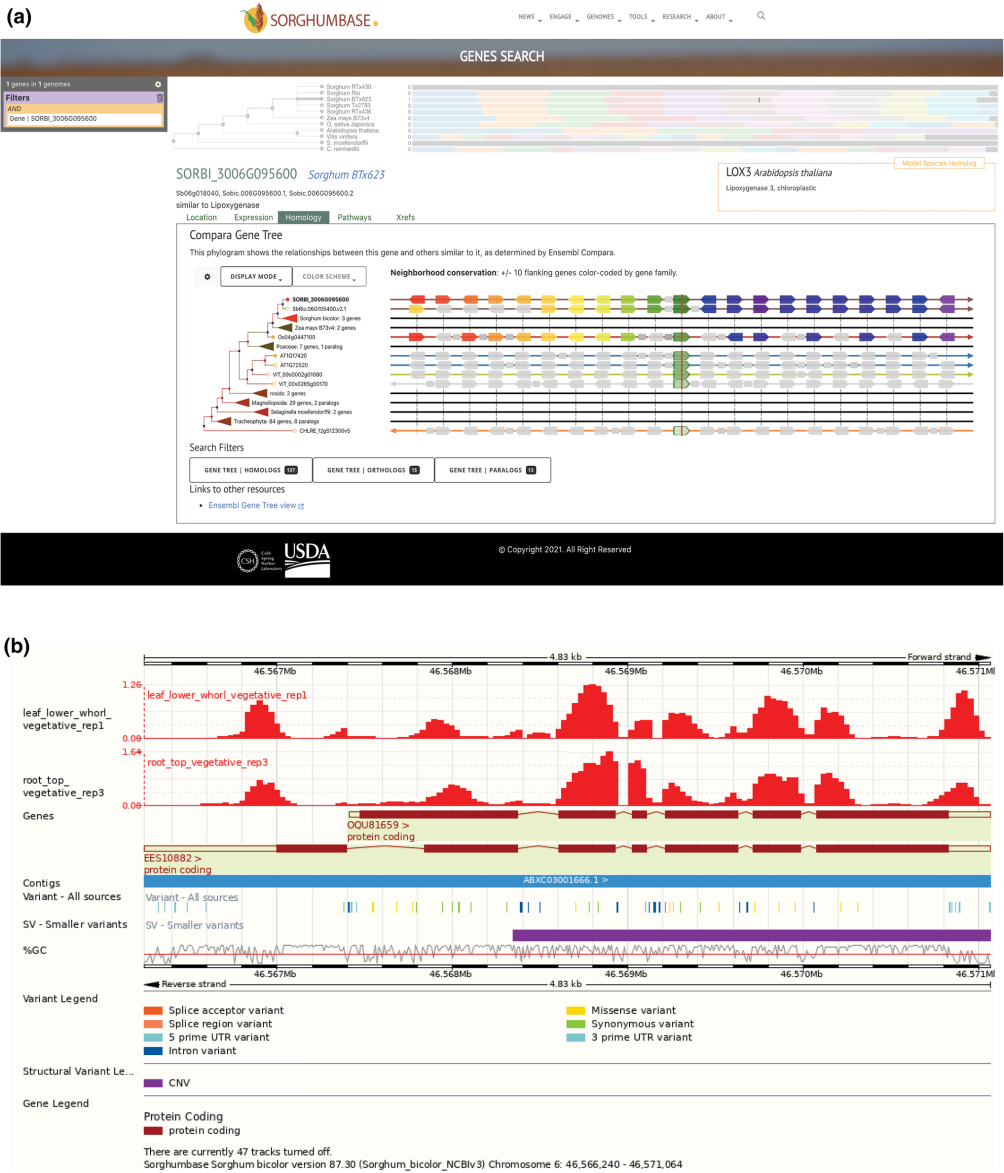
SorghumBase portal functionality. **A** Gene search results for the sorghum *MSD2* gene; result page displays gene tree views and has changeable genome features and quick-views of available expression and metabolic data. **B** Ensembl browser view displaying epigenetic marks as well as cataloged SNP and structural variants associated with the surrounding gene models

**Fig. 2:**
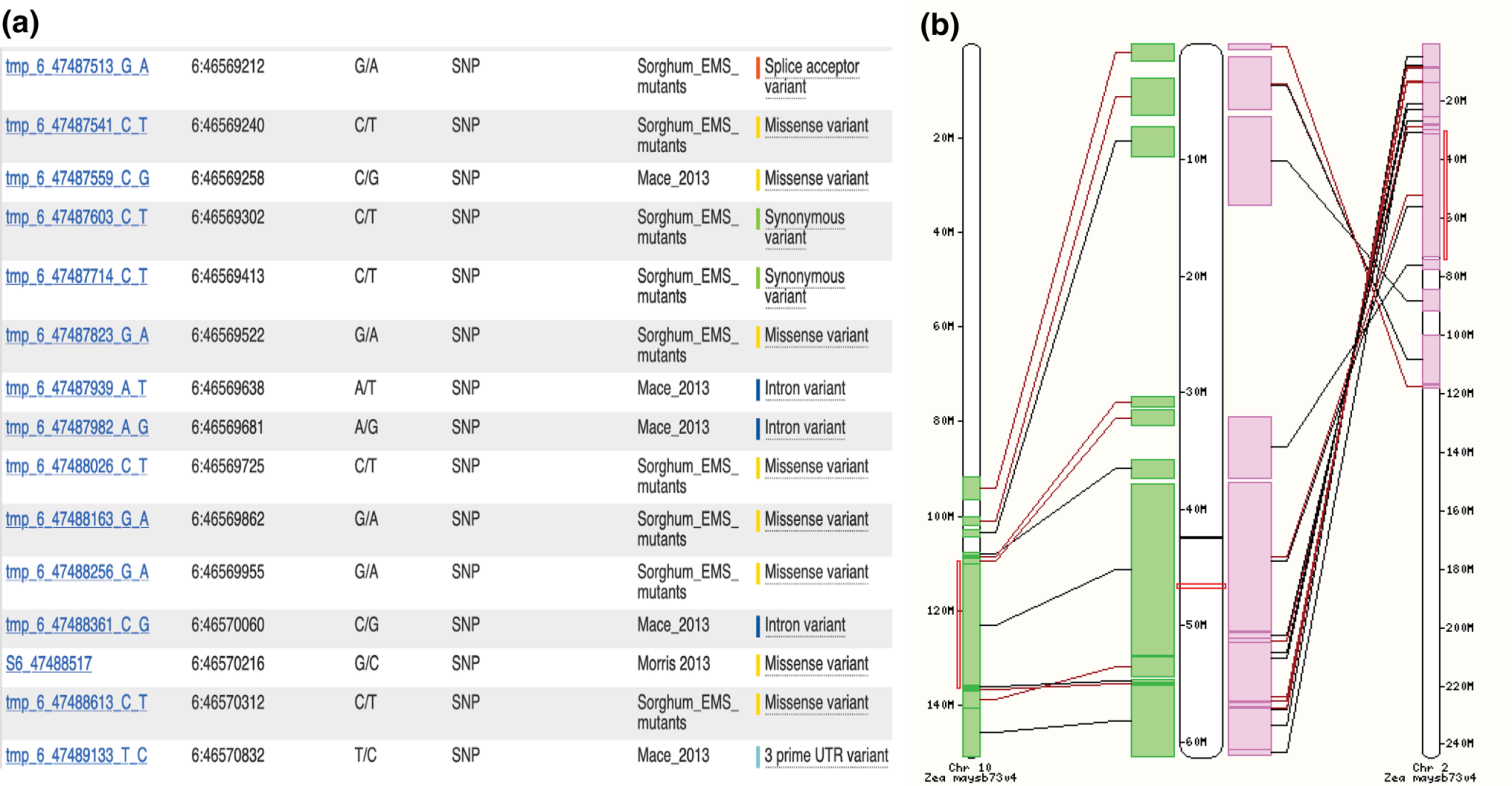
SorghumBase data visualizations. **A** Variant table displaying EMS-induced SNPs for *MSD2*, colorized for mutation consequence; the table can be filtered for SIFT score. **B** Synteny between *Sorghum* BTx623 v3 and *Zea mays* v4 chromosome assemblies

Genome cores provide the foundation for building protein-based gene trees. In Release 1, we used five sorghum genomes as inputs for the protein-based gene trees for Ensembl Protein Comparative phylogenetic analysis (version-87, https://doi.org/10.1093/database/bav096) and seven outgroups [*Arabidopsis thaliana* (TAIR10) (Berardini et al. 2015), *Oryza sativa* (IRGSP-1.0) (Kawahara et al. 2013), *Vitis vinifera* (IGGP_12x) (Jaillon et al. 2007), B73 *Zea mays* (AGPv4) (Jiao et al. 2017)], *Chlamydomonas reinhardtii* (Chlamydomonas_reinhardtii_v5.5) (Merchant et al. 2007), *Selaginella moellendorffi*i (v1.0) (Banks et al. 2011), and *Drosophila melanogaster* (BDGP6) (dos Santos et al. 2015). The resultant analyses had 21,429 protein-coding gene family trees, constructed using the peptide encoded by the canonical transcript (i.e., a representative transcript for a given gene) of each 317,845 individual genes (350,099 input proteins) from the 12 genomes. These gene trees provide the framework for phylogenomic dating of sorghum genes and establishment of orthologs and paralogs, facilitating movement between and within species as well as characterization of the species pan-gene set (Fig. 1). The gene trees provide the input for building homology views that are available from the gene search. The gene trees and position information for each gene are used to generate gene neighborhood views. In Fig. 1A, the BTx623 sorghum gene SORBI_3006G095600 is central, and the local neighborhood is expanded to ten genes on either side. Genes are color-coded based on the trees to which they belong. All five sorghum genomes have a candidate allele of the SORBI_3006G095600 homolog. The gene neighborhoods are similar: Tx2783 and Tx436 have an additional gene, indicated by the gray icon. Figure 1B demonstrates the genome browser function with various user-customizable genomic data tracks, including positions of SNPs from ethyl methanesulfonate (EMS) and natural variant populations. Figure 2A shows the available SNP populations that lie around the *MSD2* gene model; researchers can select mutations with a higher probability of deleterious impact on gene function via SIFT scores. For breeding purposes, the high degree of gene order conservation with rice and maize can be visualized through syntenic comparisons; one of the two orthologous regions of maize exhibits a lower level of conservation than the other (Fig. 2B). Expansion of the leaves on the trees reveals 12 paralogs of *MSD2* in the sorghum BTx623 genome, as seen by the paralogs tab at the display bottom.

## Sorghum genetic variation

SorghumBase contains variant data for SNPs and structural variants. Although SorghumBase is a pan-genome resource, BTx623 serves as the primary reference coordinate for calling genetic variations and pathway projections. Release 1.0 contains SNPs from natural (Mace et al. 2013; Morris et al. 2013) and EMS-induced populations (Jiao et al. 2016). These combined datasets comprise over 8.5 million SNPs covering > 85% of the initial reference genome and > 95% of gene space, creating a rich resource for forward and reverse genetic analysis. The genetic variation data are stored in an Ensembl variation database. The Ensembl variation effect predictor (VEP) (McLaren et al. 2016) is used to predict impacts on gene products through SIFT scores (Kumar et al. 2009). The impacts of the SNPs can be viewed in the genome browser tracks and are also listed by SIFT scores and color-coded in table format (Figs. 1B, 2A).

## Phenotypes

SorghumBase currently hosts two types of phenotype data: quantitative trait loci (QTLs) (Mace et al. 2019) and gene expression (Papatheodorou et al. 2020). The QTL data are directly imported from the Sorghum QTL Atlas (https://aussorgm.org.au/sorghum-qtl-atlas/) in collaboration with the University of Queensland and Research Facilities of the Department of Agriculture and Fisheries. Intended as an applied breeding resource, this platform contains data from more than 150 QTL and GWAS studies for > 200 unique traits classified into seven broad categories: stem morphology, stem composition, leaf, panicle, abiotic resistance, biotic resistance, and maturity [modified from Mace and Jordan (2011)]. In the SorghumBase first release, the QTL data are available as genome browser tracks, allowing the user to identify candidate genes underlying QTLs. A dropdown in the genome browser menu contains links back to the Sorghum QTL Atlas, providing interoperability between sorghum community resources. Gene expression data are directly imported from the EBI Gene Expression Atlas. These profiles cover 24 different tissues and abiotic stress conditions (Supplemental Table 1) (Papatheodorou et al. 2020) and can be viewed as transcripts per million (TPM) heatmaps for individually searched genes; individual experiments can also be downloaded directly from the linked EBI portal. This allows users to simultaneously visualize gene expression data for multiple tissues and rapidly winnow candidate loci.

## Public engagement, outreach, and training

We engage the community through webinars, surveys, individual exchanges, and in-person meetings. In addition, we host virtual office hours and a real-time messaging service (e.g., Slack). The SorghumBase content management system (CMS) was built using the WordPress platform. Team members and authorized contributors are able to use a simplified interface to create web pages, add content, and customize designs through the CMS dashboard. As of publication, there are five blog posts, five news items, and five research notes. In addition, the platform has a quick-start guide to orient users with existing tools and datasets (https://sorghumbase.org/guides).

## Future directions

Although SorghumBase serves as a nexus for valuable data, its continued success as a facilitator and launching point for research and collaboration ultimately relies upon the engagement of the sorghum community. Of utmost consideration are scaling genomes for future pan-genome inclusion and creating sufficient browser visualizations to enable ease of use. These efforts, in turn, are reliant on proper functional genomic annotations and amalgamation of priority genomes and trait-based data, such as core and dispensable genomes within the pan-genome, disease-resistance loci, QTL and GWAS integration, etc. Integration of these emerging data sets will accelerate insights into allelic variation and agronomically important traits. Our future plans include working closely with the community to establish rigorous standards for data cataloging and dissemination and growing the pan-genome. Prioritizing germplasm for future inclusion for the pan-genome will become crucial for researchers and stakeholders, and will be prioritized based on agricultural potential of the lines and quality of the reference assembly. We plan to establish working groups to improve gene annotation, genomic data collection, and engage community contributors to author research notes and news on the site. In addition to the quick-start guide and our first video tutorial on the SorghumBase search interface, including the homology views in the search results (e.g., gene neighborhood views), we plan to develop additional training materials on the Ensembl browser and BLAST alignment tools. Ultimately, SorghumBase is intended to morph around current community needs while accurately pursuing future projects that will capitalize on the larger arc of agricultural trends.

***Author contribution statement*** AO, SW, KC, MTR, ZL, IM, PVB, SK, and VK contributed to website construction, organization, and staging of datasets. AO and SW implemented the data visualizations. KC, IM, SK, BW, YJ, and VK assisted with the data organization. NG, JB, JC, GB, CH, YE, LZ, YJ, BW, and ZX contributed to site usability, use cases, data curation, and content inclusion. NG, AO, MTR, SW, KC, ZX, and DW drafted the manuscript. All authors reviewed the manuscript.

## Supplementary Information

Below is the link to the electronic supplementary material.

Supplementary file1 (DOCX 18 kb)

## Data Availability

All datasets described in this article have been previously published, are publicly available, and can be found linked from https://www.sorghumbase.org.
