# Supplementary material for: SorghumBase: a web-based portal for sorghum genetic information and community advancement
Source: Planta. 2022 Jan 11;255(2):35. doi: 10.1007/s00425-022-03821-6 (PMC8752523; doi:10.1007/s00425-022-03821-6)
Supplement: Supplementary file 1 — Supplementary file1 (DOCX 18 kb) [file 425_2022_3821_MOESM1_ESM.docx]

| **Supplemental Table 1. RNA-seq Datasets. Descriptions of the developmental stages and abiotic stress transcriptomic datasets visualized in the gene search results. Taken from EBI.** |  |  |  |  |  |  |
| --- | --- | --- | --- | --- | --- | --- |
| **Experiment ID** | **Experiment Title** | **Experimental Factor** | **#Assays** | **Experiment Description** | **PubMed ID** | **Publication DOI** |
| **Baseline Expression** |  |  |  |  |  |  |
| E-MTAB-5956 | RNA-sequencing of Sorghum tissues at different developmental stages (comparative transcriptome study) | developmental stage, organism part | 165 | We selected 11 tissues from sorghum reference genome line BTX623 for comparative study between Maize and sorghum. These 11 tissues were selected at different development stages at Cold Spring Harbor Laboratory upland farm, RNA were extracted, library was made and sequenced on HiSeq2500 PE125 platform at Woodbury Genome Center. | 29712755 | 10.1101/gr.227462.117 |
| E-MTAB-4021 | RNAseq profiling of C4 grass Sorghum bicolor bundle sheath and mesophyll cells | cell type | 6 | Triplicate replicates of bundle sheath and mesophyll cell RNAseq from the C4 grass Sorghum bicolor. | 27016024 | 10.1093/molbev/msw057 |
| E-MTAB-4203 | Transcription profiling by high throughput sequencing of Sorghum flag leaf tissue and emerging panicle tissue at panicle emergence | organism part | 4 | Sorghum plants (Btx623 background) were grown in a glasshouse between March and May 2014. At panicle emergence, two biological replicates of flag leaf tissue and two biological replicates of emerging panicle tissue were harvested and used for RNA extraction. Btx623 is a non-shattering Sorghum accession, thus in the future we would like to add additional samples from accessions with varying degrees of seed shattering. This dataset will be useful for future comparisons of 1) shattering individuals at these stages and 2) additional flowering developmental time points in a variety of accessions (http://www.rnaseqforthenextgeneration.org/profiles/carrie-thurber.html#research) | - | - |
| E-GEOD-98817 | Dynamics of gene expression during development and expansion of vegetative stem internodes of bioenergy sorghum | sampling site | 12 | - | - | - |
| E-CURD-25 | Gene Expression Regulation Associated with Vascularization in Sorghum bicolor | organism part | 12 | - | - | - |
| E-MTAB-4400 | Transcription profiling by high throughput sequencing of different tissues from Sorghum bicolor (BTx623) | organism part | 10 | The Poaceae family, also known as the grasses, includes agronomically important cereal crops such as rice, maize, sorghum, and wheat. Previous comparative studies have shown that much of the gene content is shared among the grasses; however, functional conservation of orthologous genes has yet to be explored. To gain an understanding of the genome-wide patterns of evolution of gene expression across reproductive tissues, we employed a sequence-based approach to compare analogous transcriptomes in species representing three Poaceae subgroups including the Pooideae (Brachypodium distachyon), the Panicoideae (sorghum), and the Ehrhartoideae (rice). Our transcriptome analyses reveal that only a fraction of orthologous genes exhibit conserved expression patterns. A high proportion of conserved orthologs include genes that are upregulated in physiologically similar tissues such as leaves, anther, pistil, and embryo, while orthologs that are highly expressed in seeds show the most diverged expression patterns. This experiment is related to E-MTAB-4401 (http://www.ebi.ac.uk/arrayexpress/experiments/E-MTAB-4401/) and E-MTAB-4402 (http://www.ebi.ac.uk/arrayexpress/experiments/E-MTAB-4402/) | 22443345 | 10.1111/j.1365-313X.2012.05005.x |
| E-MTAB-4273 | Transcription profiling by high throughput sequencing of seed, spikelet and stem tissues of Sorghum bicolor Btx623 | organism part | 9 | This study used with RNA-Seq to examine the tissue specific expression data within sorghum plants for improving the Sorghum bicolor gene annotation. We examined the RNA from tissues (spikelet, seed and stem) in Sorghum bicolor (BTx623).Total RNAs form each tissues were extracted using SDS/phenol method followed by LiCl purification | 25505007 | 10.1093/pcp/pcu187 |
| E-MTAB-3839 | RNA-seq of various Sorghum bicolor (BTx623) tissues: flowers, vegetative and floral meristems, embryos, roots and shoots | organism part | 17 | This experiment contains the subset of data corresponding to sorghum RNA-Seq data from experiment E-GEOD-50464 (http://www.ebi.ac.uk/arrayexpress/experiments/E-GEOD-50464/), which goal is to examine the transcriptome of various Sorghum bicolor (BTx623) tissues: flowers, vegetative and floral meristems, embryos, roots and shoots. Thus, we expanded the existing transcriptome atlas for sorghum by conducting RNA-Seq analysis on meristematic tissues, florets, and embryos, and these data sets have been used to improve on the existing community structural annotations. | - | 10.3835/plantgenome2013.08.0025 |
| **Differential Expression** |  |  |  |  |  |  |
| E-GEOD-128441 | Lifecycle transcriptomics of field-droughted sorghum reveals rapid biotic and metabolic responses | age, cultivar, environmental stress, organism part | 315 | - | - | - |
| E-GEOD-54705 | Comparison of transcriptomes between N-stress tolerant and sensitive genotypes | cultivar, environmental stress | 28 | Sorghum is an important cereal crop, which requires large quantities of nitrogen fertilizer for achieving commercial yields. Identification of the genes responsible for low-N tolerance in sorghum will facilitate understanding of the molecular mechanisms of low-N tolerance, and also facilitate the genetic improvement of sorghum through marker-assisted selection or gene transformation. In this study we compared the transcriptomes of root tissues from seven sorghum genotypes having different genetic backgrounds with contrasting low-N tolerance by the RNAseq deep sequencing data. Several genes were found which are common differentially expressed genes between four low-N tolerant sorghum genotypes (San Chi San, China17, KS78 and high-NUE bulk) and three sensitive genotypes (CK60, BTx623 and low-NUE bulk). RNAseq deep sequencing | 24597475 | 10.1186/1471-2164-15-179 |
| E-GEOD-30249 | RNA-Seq of Sorghum bicolor 9d seedlings in response to osmotic stress and abscisic acid | compound, organism part | 24 | This study utilized next generation sequencing technology (RNA-Seq) to examine the transcriptome of sorghum plants challenged with osmotic stress and exogenous abscisic acid (ABA) to elucidate those genes and gene networks that contribute to sorghum's tolerance to water-limiting environments with a long-term aim of developing strategies to improve plant productivity under drought. We examined the mRNA of 9 day old Sorghum bicolor (BTx623) from 2 tissue types (roots and shoots) for 2 treatments (20 uM ABA and 20% PEG) with corresponding controls (0.2M NaOH and H2O) for 27 hrs prior to harvesting, each done in triplicate biological replicates - resulting in 24 unique runs | 22008187 | 10.1186/1471-2164-12-514 |
